# Loss of HLTF function promotes intestinal carcinogenesis

**DOI:** 10.1186/1476-4598-11-18

**Published:** 2012-03-27

**Authors:** Sumit Sandhu, Xiaoli Wu, Zinnatun Nabi, Mojgan Rastegar, Sam Kung, Sabine Mai, Hao Ding

**Affiliations:** 1 Department of Biochemistry and Medical Genetics, University of Manitoba, 745 Bannatyne Avenue, Winnipeg MB R3E 0J9, Canada; 2 Manitoba Institute of Cell Biology, 675 McDermot Avenue, Winnipeg MB R3E 0V9, Canada; 3 Department of Immunology, University of Manitoba, 750 McDermot Avenue, Winnipeg MB R3E 0T5, Canada; 4 Regenerative Medicine Program, University of Manitoba, 745 Bannatyne Avenue, Winnipeg MB R3E 0J9, Canada

**Keywords:** HLTF, Mouse gene-targeting, Adenomatous polyposis coli (Apc), Intestinal adenocarcinoma, Colonic tumor or cancer, Chromosomal instability, HCT116 cells

## Abstract

**Background:**

HLTF (Helicase-like Transcription Factor) is a DNA helicase protein homologous to the SWI/SNF family involved in the maintenance of genomic stability and the regulation of gene expression. HLTF has also been found to be frequently inactivated by promoter hypermethylation in human colon cancers. Whether this epigenetic event is required for intestinal carcinogenesis is unknown.

**Results:**

To address the role of loss of HLTF function in the development of intestinal cancer, we generated *Hltf *deficient mice. These mutant mice showed normal development, and did not develop intestinal tumors, indicating that loss of Hltf function by itself is insufficient to induce the formation of intestinal cancer. On the *Apc^min/+ ^*mutant background, *Hltf^- ^*deficiency was found to significantly increase the formation of intestinal adenocarcinoma and colon cancers. Cytogenetic analysis of colon tumor cells from *Hltf *^-/-^*/Apc^min/+ ^*mice revealed a high incidence of gross chromosomal instabilities, including Robertsonian fusions, chromosomal fragments and aneuploidy. None of these genetic alterations were observed in the colon tumor cells derived from *Apc^min/+ ^*mice. Increased tumor growth and genomic instability was also demonstrated in HCT116 human colon cancer cells in which HLTF expression was significantly decreased.

**Conclusion:**

Taken together, our results demonstrate that loss of HLTF function promotes the malignant transformation of intestinal or colonic adenomas to carcinomas by inducing genomic instability. Our findings highly suggest that epigenetic inactivation of HLTF, as found in most human colon cancers, could play an important role in the progression of colon tumors to malignant cancer.

## Background

Human colon cancer is the second leading cause of cancer-related death in developed countries. About 50% of the Western population develops adenomatous polyps (a benign colon tumor) by the age of 70, and the lifetime risk for colon cancer is estimated to be 5% [1]. The formation of colon cancer involves a multiple-step process, starting from a small adenomatous polyp and followed by the development of a large adenoma with dysplasia that ultimately leads to the formation of invasive carcinoma (see the recent review by Fearon ER [2]). It is widely accepted that most human colon cancers are initiated by the inactivation of the Adenomatous Polyposis Coli (APC)/Wnt signaling pathway and then progress as the result of a series of mutational activation of oncogenes coupled with the inactivation of tumor-suppressor genes [2,3]. Apart from genetic mutations, epigenetic alterations, particularly aberrant CpG island methylation, have been demonstrated as a major alternative mechanism for suppressing gene function during the development of colon cancer [4-6]. To date, many genes that are epigenetically silenced in human colon cancers as well as in colonic adenomas have been identified. However, the function of many of these genes in colon carcinogenesis is still largely unknown. In this study, we have characterized the role of one of these methylated genes, termed Helicase-like Transcription Factor (HLTF), in intestinal carcinogenesis.

HLTF (SMARCA3 in OMIM) is homologous to the SWI/SNF family of chromatin remodelers [7-10]. Although HLTF was originally identified as a DNA-binding protein that could interact with several gene promoters and enhancers [7-11], recent studies indicate that this DNA helicase is more involved in the DNA-damage repair pathway. First, HLTF has been shown to exhibit an E3 ubiquitin ligase activity for the polyubiquitination of proliferating cell nuclear antigen (PCNA), which is required for the initiation of an error-free replication through DNA damage lesions [12,13]. Second, HLTF has also been found to display a double-stranded DNA translocase activity, which promotes the resolution of stalled replication forks at DNA damage lesions [14,15]. Third, a recent study indicates that HLTF also possesses a chromatin remodeling activity, which leads to the displacement of DNA-bound proteins on stalled replication forks and facilitates DNA-damage repair [16]. These findings demonstrate that HLTF may be a functional homologue of yeast rad5 and that it plays an important role in an error-free post-replicative repair pathway. The requirement of HLTF for repair of damaged DNA may also implicate a tumor suppression role in human colon cancers, where HLTF has been identified as a common target for methylation and epigenetic gene silencing.

Epigenetic inactivation of HLTF gene expression by promoter hypermethylation has been reported in more than 40% of human colon cancers [17-20]. The frequency of *HLTF *promoter methylation was found to increase drastically between early stage of adenomas and advanced adenomas, suggesting that this epigenetic alteration could be a later event in colon carcinogenesis [17,19]. In addition, *HLTF *methylation was demonstrated to be significantly correlated with poor prognosis in human colon cancer patients [21,22]. Besides colon tumors, *HLTF *methylation was also commonly detected in human gastric tumors or cancers, but not in other human cancers, such as lung and breast cancers [17,23-25], suggesting that this epigenetic alteration is unique to the tumors of the gastrointestinal tract. However, although *HLTF *methylation has been demonstrated as a common event in human colon cancer and it has been suggested as a prognostic biomarker [22,26], it is largely unclear whether this epigenetic event is important for the development of this cancer. In the present study, we generated an *Hltf *deficient mouse allele, and applied this mouse model to characterize the role of loss of HLTF function in the development of intestinal cancer. Our data demonstrates that loss of Hltf has a promoting effect on the transition of intestinal adenomas to invasive cancers, which highly suggests that aberrant HLTF methylation, as found in most human colon cancers, may have an important pathogenetic role in the development of this cancer.

## Results

### Generation of the *Hltf *null mouse allele

**Figure 1:**
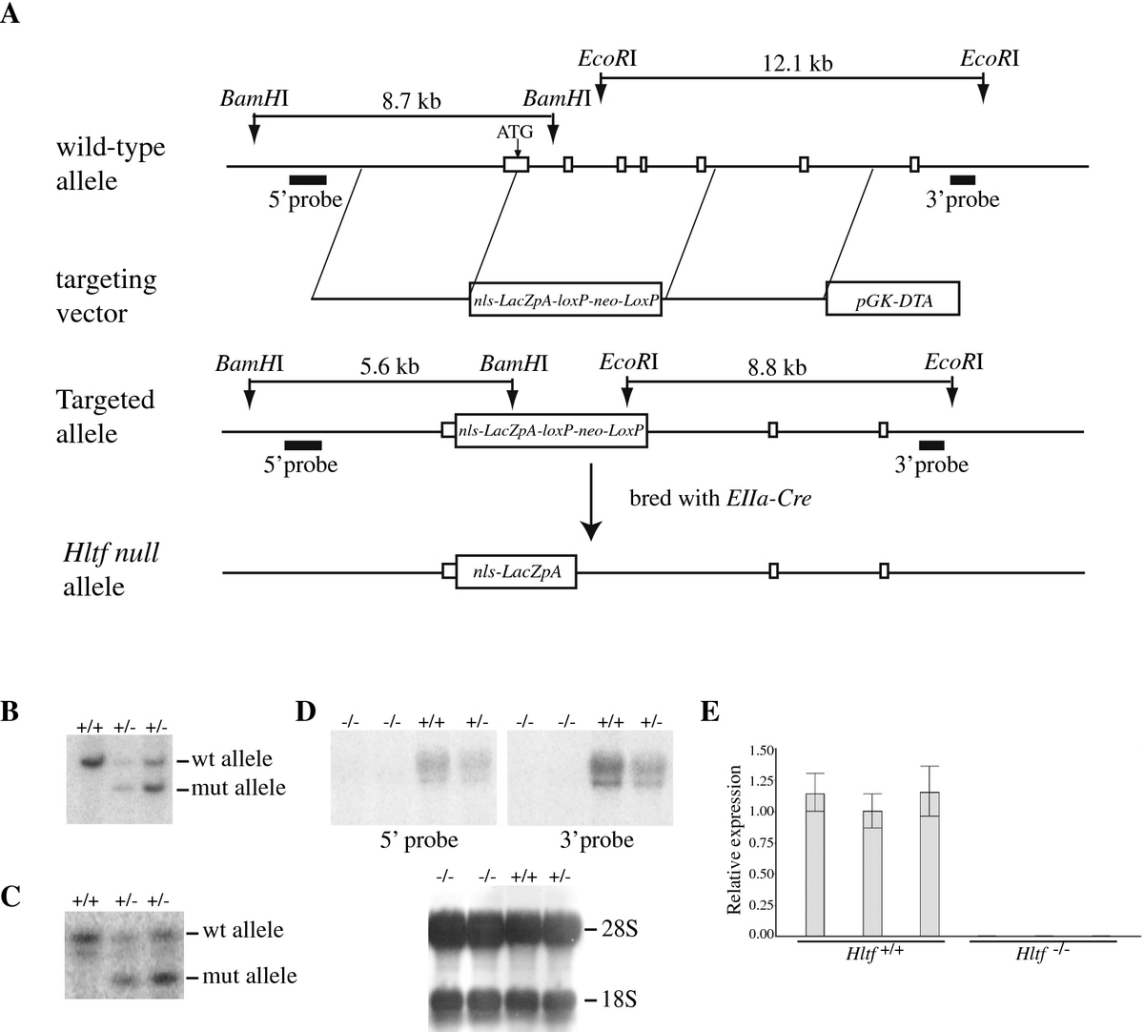
**Targeted disruption of *Hltf *in mice by homologous recombination**. (A) Schematic representation of the first 7 exons of the mouse *Hltf *locus, the gene-targeting vector, and the mutant alleles. The location of the hybridization probes (5' probe and 3' probe) for Southern blot analyses are shown. (B) Southern blot analysis of targeted ES cell clones using 5' probe. The genomic DNA was digested with *Bam*H1 and hybridized with the 3' probe, giving a wild-type allele of 8.7 kb and a mutant allele of 5.6 kb. (C) Southern blot analysis with the 3'probe, showing the targeted allele with a size of 8.8 kb and the wild-type allele of 12.1 kb fragment upon *Eco*RI digestion. (D) Northern blot analysis of *Hltf *transcripts in the E10.5 embryos of the indicated genotypes using cDNA probes that cover 5' and 3' coding sequence of *Hltf*, respectively. The 28S and 18S ribosomal RNAs are shown as loading controls. (E) Real-time RT-PCR for detecting the 3' coding sequence of *Hltf *in derived *Hltf *^-/- ^ES cells. The presented relative expression was normalized to the expression of *Gapdh*. No *Hltf *transcripts were detected in *Hltf *^-/- ^samples by both Northern blot and real-time RT-PCR assays.

To study the loss of function of Hltf *in vivo*, we mutated *Hltf *in mice by homologous recombination. A gene-targeting vector was generated to replace the exons 1-5 coding for residues 2-208 of Hltf with a nuclear localized *LacZ *(*nls-LacZ*) cDNA that was fused in-frame with the endogenous *Hltf *starting codon (Figure 1A). Correct homologous recombination in the targeted ES cells was confirmed by Southern blot analysis using both 5' and 3' probes located upstream and downstream of the targeted *Hltf *sequence (Figure 1B and 1C). Two independent ES clones were used to produce germline-transmitting chimeras that were further bred with a ubiquitous Cre transgenic line (*EIIa-Cre*) to delete the *lox*P flanked *pGK-neo *selection marker from the targeted locus (Figure 1A). The resultant *Hltf *^+/- ^mouse, which was maintained on either a C57BL/6 or C57BL/6/129S1 background, was used to produce *Hltf *homozygous mutant mice for phenotypic characterization.

To determine whether Hltf expression is completely inactivated in *Hltf *^-/- ^mice, we performed Northern blot analysis of total RNA prepared from control and mutant E10.5 embryos. Whereas *Hltf *transcripts were identified in control (wild-type and *Hltf *^+/-^) embryos using the 5' and 3' *Hltf *cDNA probes, transcripts were not detected in *Hltf *^-/- ^samples (Figure 1D). To further demonstrate the absence of *Hltf *expression in *Hltf *^-/- ^mice, we also applied a more sensitive real time RT-PCR assay to examine the expression of *Hltf *in derived *Hltf *^-/- ^mouse embryonic stem (ES) cells or mouse embryonic fibroblast (MEF) cells as well as in *Hltf *^-/- ^E10.5 embryos. With the primer set located on the 3' of *Hltf *cDNA, a strong amplification of *Hltf *was detected in the control group, but no signal was found in *Hltf *^-/- ^samples (Figure 1E). These data clearly indicate that the targeted mutation that we introduced into the *Hltf *mouse genomic locus gave rise to a null allele.

### Normal development and lack of intestinal tumors in *Hltf *null mice

**Figure 2:**
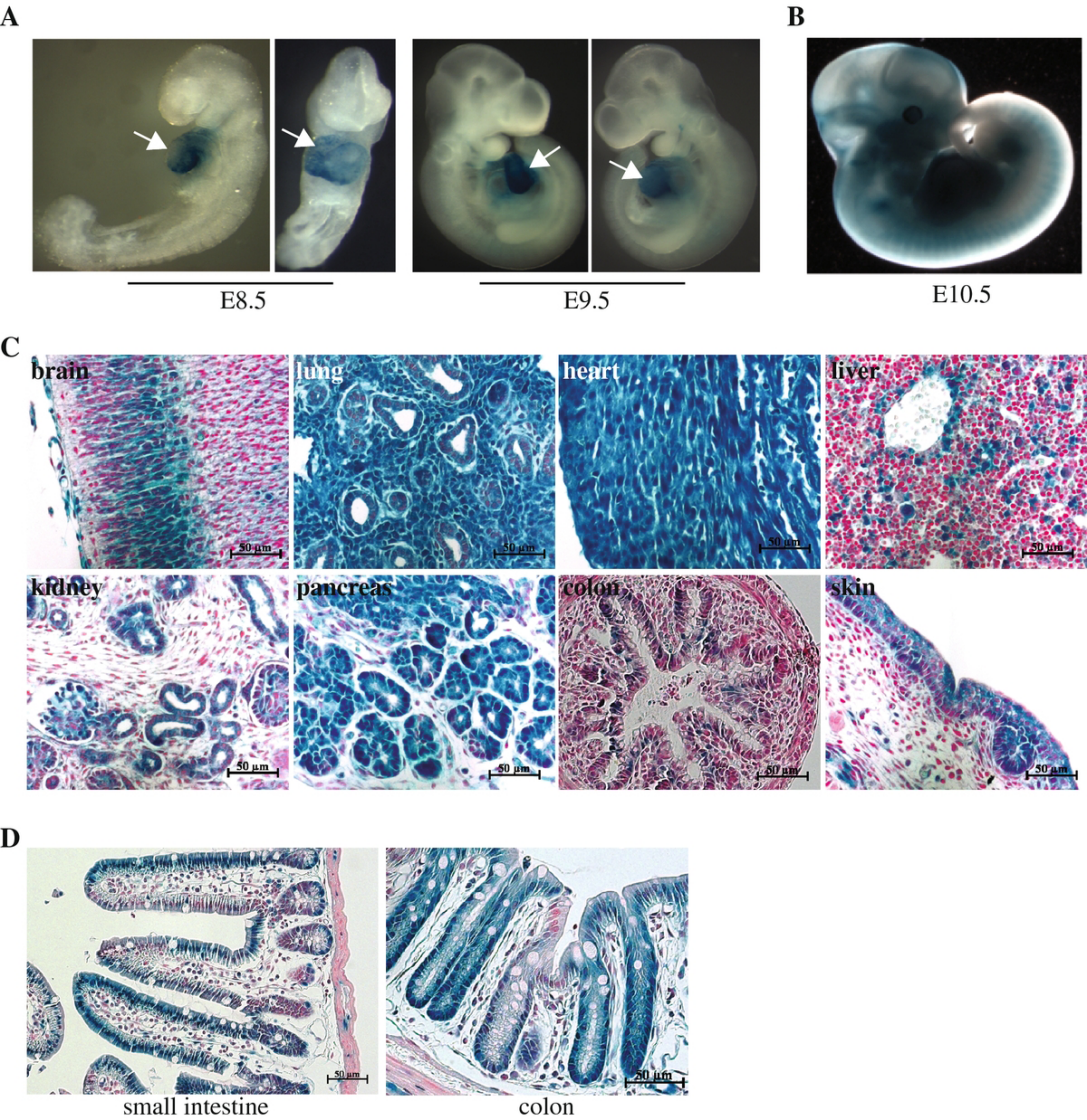
**Expression of *Hltf *during mouse development**. The analysis was done by determining the activity of LacZ in *Hltf *knockout mice in which the *lacZ *reporter was fused in-frame with the endogenous *Hltf *start codon. (A) Whole mount X-gal staining of E8.5 and E9.5 *Hltf *^+/- ^embryos, demonstrating the specific expression of *Hltf *in heart (arrows indicate). (B) Whole mount X-gal staining of E10.5 *Hltf *^+/- ^embryos shows broad expression of *Hltf*. (C) X-gal staining of tissues collected from E16.5 *Hltf *^+/- ^embryos. *Hltf *was found to be widely expressed in many tissues at this developmental stage. (D) X-gal staining of intestine and colon from two-month old *Hltf *^+/- ^mice. *Hltf *was identified to be predominately expressed in the crypts as well as in the intestinal epithelial cells in both intestine and colon.

With the established *Hltf *null allele, we then asked whether loss of Hltf function could affect mouse development and induce the formation of intestinal tumors. As a first approach to address this, we determined the expression pattern of Hltf during development. We took advantage of the inserted *nls-LacZ *reporter gene in *Hltf *knockout mice, whose expression is controlled by the endogenous *Hltf *regulatory elements. Both *Hltf *^+/- ^and *Hltf *^-/- ^mice showed the same LacZ expression pattern. By determining LacZ activity in these mice, we found that *Hltf *was specifically expressed in the heart at an early developmental stage (E8.5 to E9.5) (Figure 2A). *Hltf *exhibited a broader expression pattern at E10.5, with LacZ signals detected in somites, branchial arches, limb bud and brain (Figure 2B). At later embryonic developmental stages, such as E16.5, *Hltf *showed wide and strong expression in many tissues, including heart, lung, liver, kidney, spleen and pancreas (Figure 2C). This wide-spread expression of *Hltf *was also observed in adult mice (data not shown). In both adult intestine and colon, *Hltf *expression was mainly detected in the crypts and in the intestinal epithelial cells (Figure 2D).

This broad expression pattern of Hltf during mouse development might suggest that Hltf is important for development. However, *Hltf *^-/- ^mice were found to have normal embryonic and postnatal development. The *Hltf *null mice were fertile, and exhibited similar a body weight and life span as their wild-type (wt) littermates. In addition, *Hltf *^-/- ^mice did not display obvious abnormalities of intestinal proliferation or differentiation as determined by the BrdU incorporation assay as well as by the analyses with different intestinal cell lineage markers (Additional file 1 and Additional file 2). To determine whether loss of Hltf function could induce the formation of intestinal tumors, we monitored a cohort of 60 mice fed with a normal diet for a two-year period (30 *Hltf *^-/- ^mice on C57BL/6 background and 30 wt mice matched for age and genetic background). Similar to the wt control group, 10% of *Hltf *^-/- ^mice (3 out of 30) developed liver tumors or lymphomas between 16 and 24 months. However, none of *Hltf *^-/- ^mice formed intestinal tumors or gastric tumors.

Taken together, our results demonstrate that Hltf is dispensable for normal development. Our work also indicates that loss of Hltf function by itself is insufficient to drive the oncogenic process in the gastrointestinal tract, which implicates that the epigenetic inactivation of HLTF, as commonly found in human colon cancers, could be involved in the late stage of intestinal tumor development.

### Development of intestinal cancers in *Hltf^-/-^/Apc^min/+ ^*mutant mice

To determine whether the loss of HLTF function could have a role in the progression of intestinal tumors, we introduced the *Hltf *null mutation into *Apc^min/+ ^*mice. The *Apc^min/+ ^*mice harbor a premature stop codon in one allele of the *Apc *tumor suppressor gene, and develop multiple intestinal adenomas that mimic human familial adenomatosis polyposis [27]. The adenomas in *Apc^min/+ ^*mice rarely progress to invasive intestinal or colon cancers [28], making this mouse model an excellent genetic tool for studying the later events involved in intestinal carcinogenesis.

**Figure 3:**
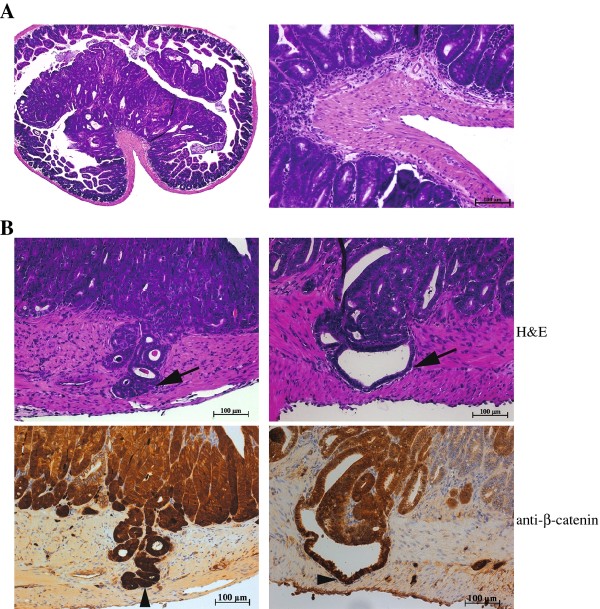
**Loss of Hltf function enhances the formation of intestinal adenocarcinomas on Apc mutant background**. (A) Haematoxylin-eosin staining of representative intestinal tumors developed in *Apc^min/+ ^*mice, showing the formation of a well-defined tubular adenoma within the lamina propria in these mice. (B) Two representative intestinal adenocarcimas from *Hltf *^-/-^*/Apc^min/+ ^*mice. Arrowhead indicates tumor invasion through the submucosa and into the muscularis propria. Immuno-staining with anti-β-catenin antibody also demonstrated that the invasive neoplatic glands contained high β-catenin activity as reflected by the nuclear accumulation of β-catenin signals (indicated by arrowheads)

To avoid strain-specific modification of the *Apc^min/+ ^*intestinal tumor phenotype [29,30], we generated *Hltf *^-/-^/*Apc^min/+ ^*mice on the C57BL/6 background. *Hltf *^-/-^/*Apc^min/+ ^*mice became moribund at a similar age as *Apc^min/+ ^*mice. Post-mortem examination revealed that *Hltf *^-/-^/*Apc^min/+ ^*and *Apc^min/+ ^*mice developed similar numbers of tumors in the small intestine (average of 65 macroscopic tumors/per mouse in both groups). However, upon histological characterization, most *Hltf *^-/-^/*Apc^min/+ ^*mice (24 out of 30) were found to form invasive intestinal adenocarcinomas characterized by deeper invasion of tumor cells into the muscularis propria (Figure 3B and Additional file 3). The invaded neoplastic glandular cells exhibited high β-catenin activity as reflected by the nuclear accumulation of β-catenin detected by immmuno-staining with anti-β-catenin antibody (Figure 3B). In contrast, most *Apc^min/+ ^*mice developed only well-defined tubular adenomas or adenomas within the lamina propria (Figure 3A).

**Figure 4:**
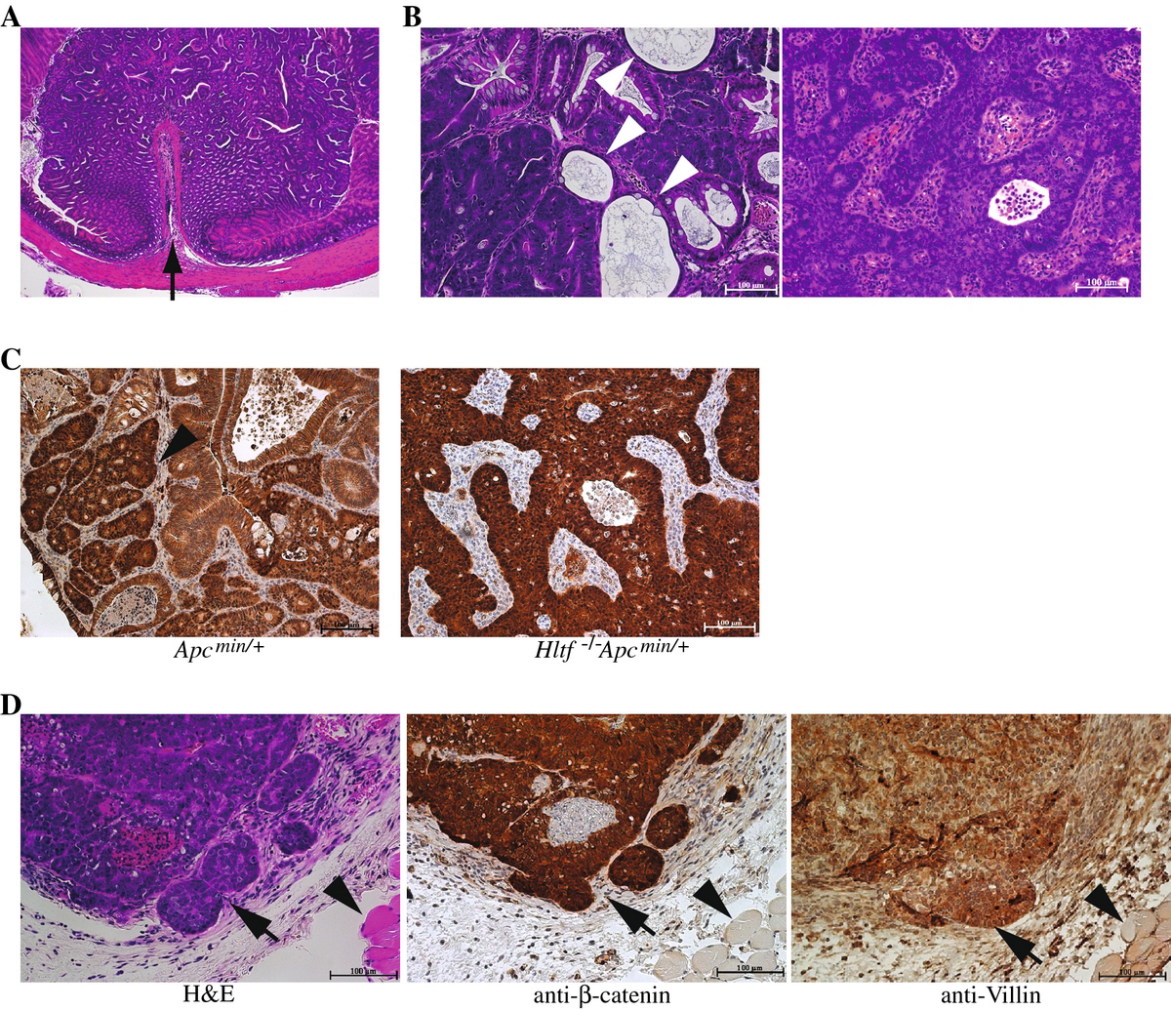
***Hltf*^-/-^*/Apc^min/+ ^*mice develop malignant colon tumors that are transplantable into another host**. (A) Haematoxylin-eosin staining of representative colon tumors collected from *Apc^min/+ ^*mice, showing pronounced tumor growth a pedunculated morphology protruding into the colonic lumen (indicated by arrow). (B) Representative colon tumors collected from *Hltf *^-/-^*/Apc^min/+ ^*mice, demonstrating the formation of numerous mucin-filled cysts within tumors (white arrowheads) and strong desmoplastic stromal reaction. (C) Immuno-staining with anti-β-catenin antibody. *Hltf *^-/-^*/Apc^min/+ ^*colon tumors had stronger and more ubiquitous nuclear-stained β-catenin signals than the tumor cells in *Apc^min/+ ^*mice. Arrowheads indicate the tumor cells with nuclear-stained β-catenin. (D) The formation of subcutaneous tumors from derived *Hltf *^-/-^*/Apc^min/+ ^*colon tumor cells in *Rag1*^-/-^*/IL2*^-/- ^immunodeficient mice. The subcutaneous tumors displayed neoplastic glandular structures, which contained strong nuclear-stained β-catenin signals and were also positive for Villin, a marker for intestinal epithelial cells. Arrows indicate the tumor, whereas arrowheads mark the subcutaneous muscle layer.

*Hltf *^-/-^/*Apc^min/+ ^*mice were also found to develop a similar number of neoplastic lesions in the colon as *Apc^min/+ ^*mice (average of 1.5 macroscopic tumors/per mouse). Histologically, although both *Hltf *^-/-^/*Apc^min/+ ^*and *Apc^min/+ ^*colon tumors showed tumor growth with a pedunculated morphology protruding into the colonic lumen, the majority of *Hltf *^-/-^/*Apc^min/+ ^*colon tumors displayed a more dramatic glandular atypia that resulted in the formation of numerous mucin-filled cysts (Figure 4A, B), a characteristic pathological feature for human colon cancers [31]. In addition, most *Hltf *^-/-^/*Apc^min/+ ^*colon tumors were also found to have a strong desmoplastic stromal reaction, suggesting invasion of the lamina propia (Figure 4B). Furthermore, *Hltf *^-/-^/*Apc^min/+ ^*colon tumors were also found to have stronger and more ubiquitous nuclear-stained β-catenin signals than the tumor cells in *Apc^min/+ ^*mice (Figure 4C). These data indicate that colon tumors from *Hltf *^-/-^/*Apc^min/+ ^*mice had progressed to much higher grades than those from *Apc^min/+ ^*mice. To further demonstrate the malignancy of colon tumors developed in *Hltf *^-/-^/*Apc^min/+ ^*mice, we derived cells from these tumors and then subcutaneously injected them into *Rag1*^-/-^*/IL2*^-/- ^immunodeficient mice. Within 30 days after injection, these cells formed subcutaneous tumors that maintained the morphological and immunohistochemical features of the parental colon tumors (Figure 4D). This capability of propagating tumor cells into another host, which was not observed with cells derived *Apc^min/+ ^*colon tumors, further supports that many colon tumors developed in *Hltf *^-/-^/*Apc^min/+ ^*mice could be malignant cancers.

Collectively, our results demonstrate that loss of Hltf function is able to promote intestinal or colonic carcinogenesis in mice with a mutant Apc background. This finding, together with human epigenetic evidence that HLTF is frequently silenced in the advanced human colon adenomas or cancers but uncommonly in early adenomas [17,19], indicates that epigenetic inactivation of HLTF could be an important event involved in the transition of benign adenomas to malignant colon cancer.

### Chromosomal instability in *Hltf^-/-^/Apc^min/+ ^*colon tumor cells

Chromosomal instability (CIN) is a genetic hallmark of most human colon cancers, and it has been demonstrated to be important for the progression of colon tumors by increasing the rate of genetic aberration [2,32,33]. Since HLTF has been shown to be required for the maintenance of genomic stability [12-16], it is likely that intestinal or colonic tumors developed in *Hltf *^-/-^/*Apc^min/+ ^*mice would have increased genomic instability. To examine this, we analyzed metaphase spreads generated from tumor cells (passage 2) derived from colon tumors developed in *Apc^min/+ ^*and *Hltf *^-/-^/*Apc^min/+ ^*mice. These tumors all showed pronounced cribiform tumor growth without invasive neoplastic glands and were histologically characterized as late stage colonic adenomas.

**Figure 5:**
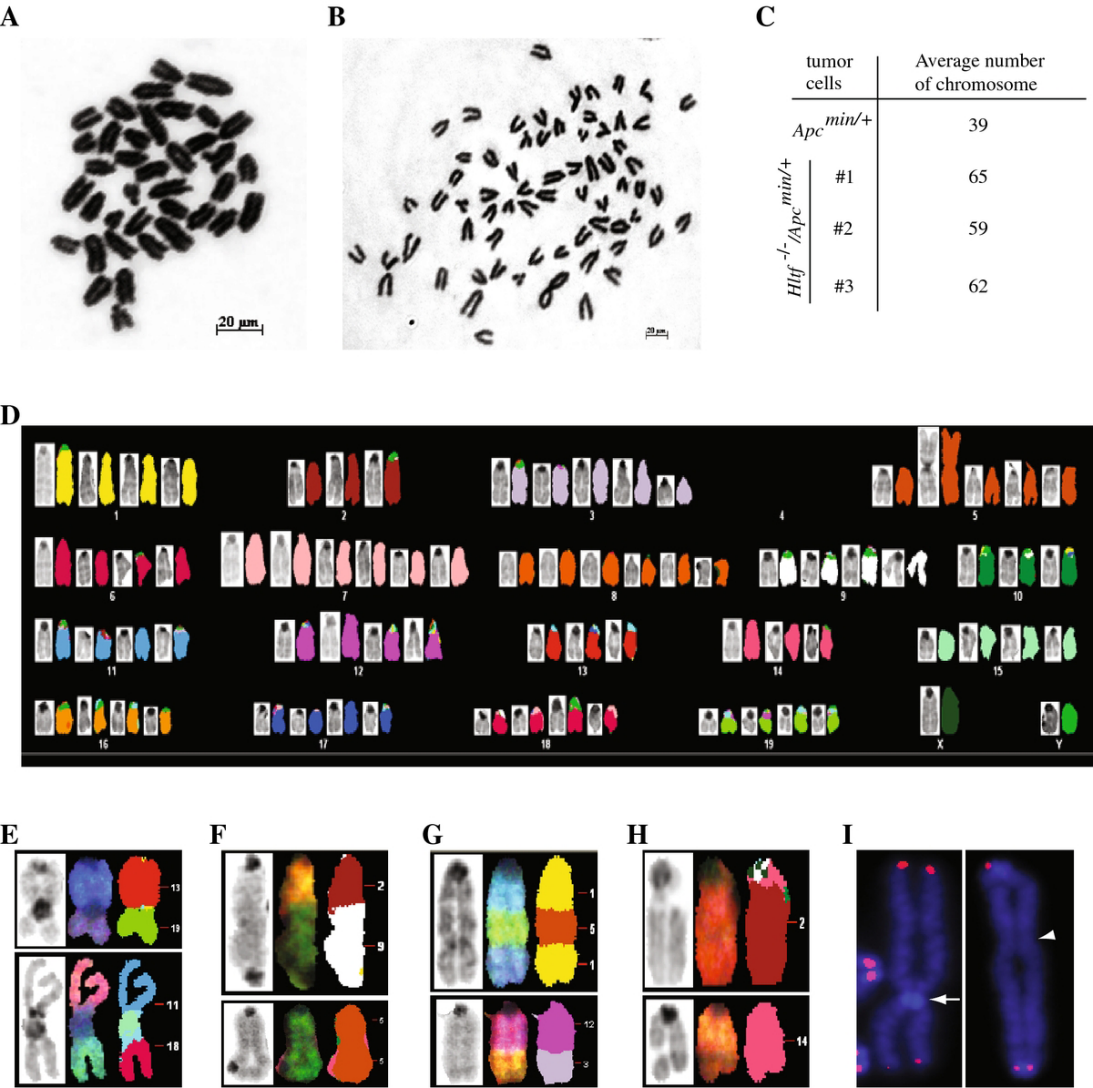
**Gross chromosomal instabilities in *Hltf^-/-^/Apc^min/+ ^*colon tumors as revealed by cytogenetic analysis**. (A) Representative metaphase spread of *Apc^min/+ ^*colon tumor cells as revealed by Giemsa staining, showing a near-diploid karyotype without obvious chromosomal abnormalities in these tumor cells. (B) Giemsa staining of metaphase spreads prepared from *Hltf *^-/-^*/Apc^min/+ ^*colon tumor cells, which displayed aneuploid and gross chromosomal instability. (C) Summary of the average number of chromosomes in derived colon tumor cells from *Apc^min/+ ^*and *Hltf *^-/-^*/Apc^min/+ ^*mice. (D) Representative spectral karyotyping (SKY) image from *Hltf *^-/-^*/Apc^min/+ ^*colon tumor cells. (E-I) Some commonly detected chromosomal abnormalities in *Hltf *^-/-^*/Apc^min/+ ^*colon tumor cells. E: Robertsonian fusions; F: Dicentric chromosomes; G: Unbalanced chromosomal translocations; H: Chromosomal breaks. I: Telomere dysfunction as revealed by Q-FISH. A white arrow indicates chromosomal fusion without telomeres at the fusion site, and a white arrowhead points to lack of telomere signals at the fusion site of a dicentric chromosome ring.

Consistent with previous findings [34,35], colon tumor cells from *Apc^min/+ ^*mice showed a near-diploid karyotype and did not harbor obvious chromosomal abnormalities (Figure 5A). In contrast, three colon tumor cell lines derived from *Hltf *^-/-^/*Apc^min/+ ^*mice displayed an aneuploid phenotype (Figure 5B and 5C). Spectral karytotype (SKY) analysis on these tumor cells further revealed a high incidence of genetic alterations in the forms of nonreciprocal translocations, chromosomal fusions (which include both dicentric chromosomes and centromeric fusions), and chromosomal fragments and breaks (Figure 5D-G and Table 1). In addition, many chromosomal fusions in *Hltf *^-/-^/*Apc^min/+ ^*colon tumor cells were found to lack telomere signals (Figure 5I), indicating the presence of telomere dysfunction in these tumor cells. Collectively, these results demonstrate that *Hltf *^-/-^/*Apc^min/+ ^*colon tumors had gross chromosomal abnormalities, similar to the CIN phenotype as described in most human colorectal cancers.

**Table 1:** Summary of cytogenetic aberrations observed in colon tumors from *Hltf ^-/-^Apc*^min/+ ^mice

Genotype of tumors	Metaphase Analyzed	Aneuploidy (%)^a^	Translocations (%)	End-to-End Fusions (%)^b^	Fragments (%)	Breaks (%)
*Apc^min/+^*	20	3 (15%)	0	0	0	0

*Hltf^-/-^/Apc^min/+^*	20	17 (85%)	7 (35%)	9 (45%)	16 (80%)	3 (15%)

*Hltf^-/-^/Apc^min/+^*	33	31 (93%)	10 (32%)	21 (63%)	15 (50%)	4 (20%)

*Hltf^-/-^/Apc^min/+^*	20	20 (100%)	3 (15%)	4 (20%)	1 (5%)	4 (20%)

^a ^Expressed as percentage of cells with other than 40 chromosomes (each Robertsonian fusion counted as two chromosomes).

^b ^Expressed as total number of end-to-end associated chromosomes per metaphase, including dicentric chromosomes, telomere associations and Robertsonian fusions

### Increased tumor growth and chromosomal instability in HCT116 human colon cancer cells with HLTF knockdown

**Figure 6:**
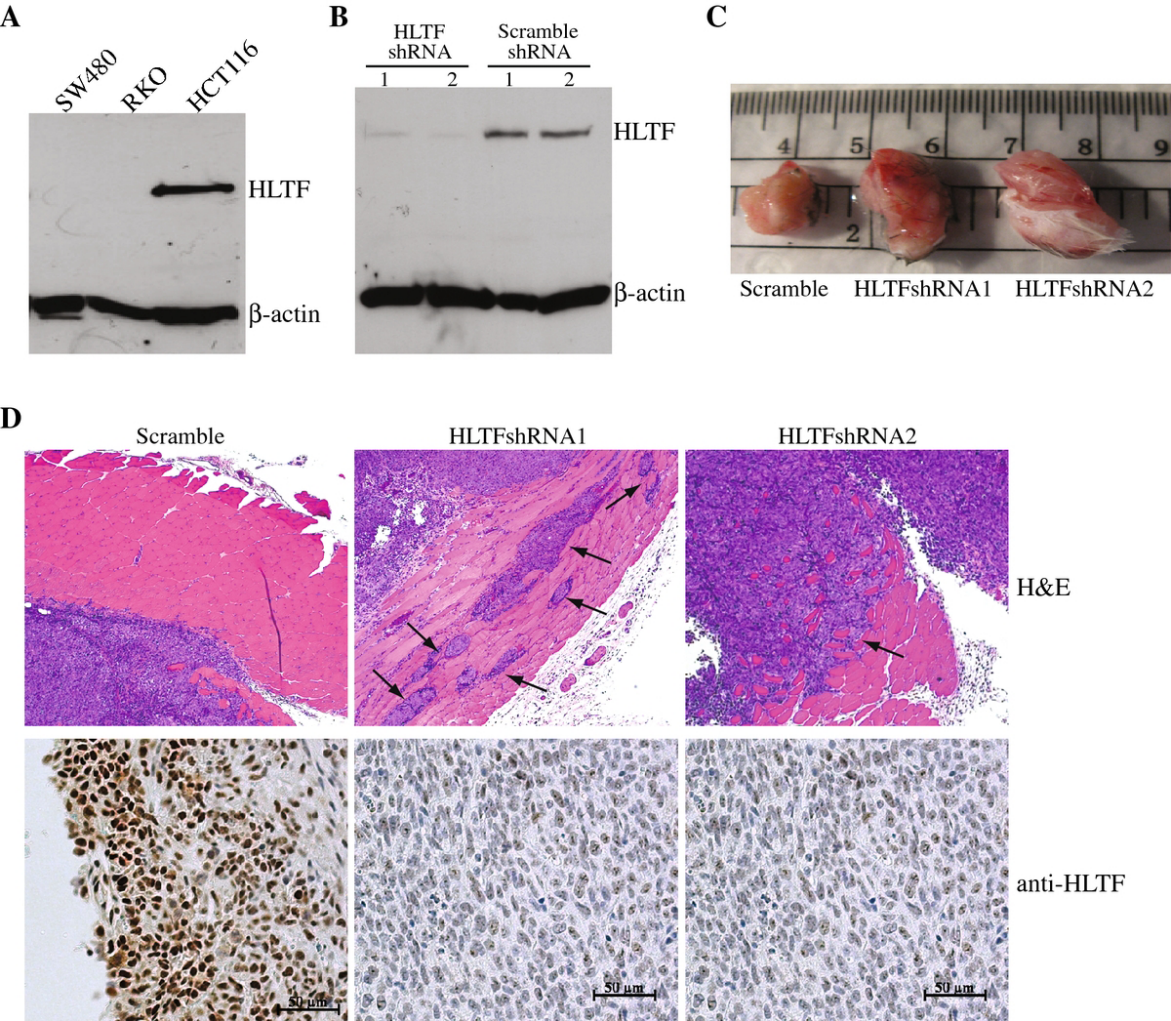
**Down-regulation of HLTF in human HCT116 colon cancer cells promotes tumor growth**. (A) Western blot analysis with anti-HLTF antibody on the cell lysates prepared from *HLTF *methylated human colon cancer cells (RKO and SW480) and *HLTF *unmethylated human colon cancer cells (HCT116). (B) Western blot analysis on the cell lysates prepared from HCT116 cells that were stably integrated with scramble control shRNA and *HLTF *shRNAs. HLTF expression was efficiently inhibited by two independent HLTF shRNAs. (C) Representative image of subcutaneous tumors formed from HCT116^Scramble^, HCT116^HLTFshRNA1 ^and HCT116^HLTFshRNA2 ^cells in *Rag1*^-/-^*/IL2*^-/- ^immunodeficient mice. (D) Haematoxylin-eosin staining demonstrates that both HLTF knockdown tumors displayed tumor cell invasion into the muscle layer (indicated by arrows) which was not typically observed in the scramble control tumors. HLTF knockdown tumors also showed low expression of HLTF as detected by immuno-staining with anti-HLTF antibody.

To determine whether loss of HLTF function would also lead to a similar tumor-promoting effect in human colon cancer as we demonstrated in the *Hltf *^-/-^/*Apc^min/+ ^*mouse model, we applied a lentiviral based shRNA knockdown approach to down-regulate HLTF expression in HCT116 human colon cancer cells. HCT116 cells are one of a few colon cancer cell lines that lack *HLTF *promoter methylation [17], and express high levels of HLTF (Figure 6A). In addition, these cells are near-diploid with a stable karyotype [36], which would also allow us to determine whether loss of HLTF function in human colon cancer cells could induce chromosomal abnormalities in a manner similar to the *Hltf*-deficient mouse colon tumor cells.

As shown in Figure 6B, HLTF expression was significantly reduced to almost undetectable protein level in the transduced HCT116 cells that were stably integrated with HLTF shRNAs (HCT116^HLTFshRNA1 ^and HCT116^HLTFshRNA2^) as compared to the scramble shRNA control (HCT116^Scramble^). To determine whether this down-regulation of HLTF expression was able to modulate HCT116 tumor growth, HCT116 cells with or without HLTF knockdown were injected subcutaneously into *Rag1*^-/-^*/IL2*^-/- ^immunodeficient mice. All the mice with injected tumor cells (10^5 ^cells/per mouse) developed subcutaneous tumors within 20 days. However, the tumors formed by HCT116^HLTFshRNA1 ^or HCT116^HLTFshRNA2 ^cells showed significantly increased tumor size as compared with that of the scramble control (Figure 6C). Moreover, both HCT116^HLTFshRNA1 ^and HCT116^HLTFshRNA2 ^subcutaneous tumors also frequently exhibited the invasion of tumor cells into the subcutaneous muscle layer that was uncommonly observed in the control tumors (Figure 6D). To confirm that HLTF expression was indeed down-regulated in these tumors, we performed immunohistochemistry with an anti-HLTF antibody. Both HCT116^HLTFshRNA1 ^and HCT116^HLTFshRNA2 ^subcutaneous tumors showed significantly lower expression of HLTF as compared to the scramble control tumors (Figure 6D). Taken together, our data indicate that down-regulation of HLTF in human colon cancer cells may promote tumor growth and malignancy, similar to what we found in the *Hltf *^-/-^/*Apc^min/+ ^*mouse model.

**Figure 7:**
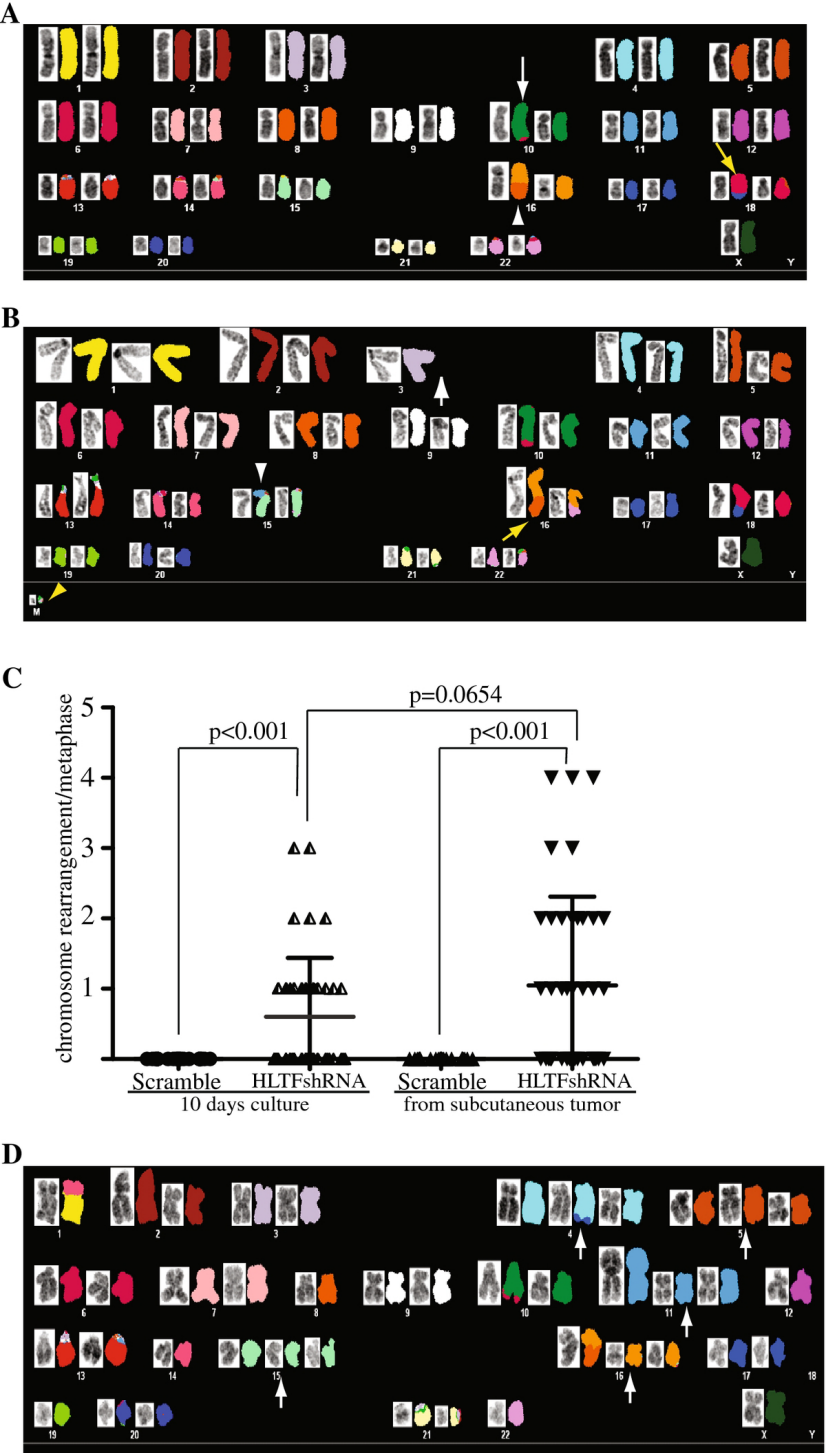
**HLTF knockdown induces chromosomal abnormalities in HCT116 colon cancer cells**. (A) Representative SKY image of HCT116^scramble ^cells, showing a stable karyotype with 45 total chromosomes, der(10)t(10;6) (white arrow), der(16)t(8;16) (white arrowhead) and der(18)t(17;18) (yellow arrow), a documented signature for HCT116 parental cells. (B) Representative SKY images of HLTF knockdown HCT116 cells. Several additional chromosomal abnormalities were identified, which include chromosomal fragment (yellow arrowhead), monosomy chromosome 3 (white arrow), der(15) (15;11) (white arrowhead) and der(16) (16;22) (yellow arrow). (C) The number of structural chromosomal abnormalities in HCT116 cells with and without HLTF knockdown. Two groups of cells were analyzed, one from a short term *in vitro *culture (10 days) and another from subcutaneous tumors that were generated by these cells. The horizontal bars indicate the mean numbers of chromosomal abnormalities in each population of cells. Each data point represents the total number of structural chromosomal rearrangements per metaphase spread after subtraction of the rearrangements (i.e. stable present translocations) found in HCT116 parental cells. (D) Representative SKY images of HLTF knockdown HCT116 cells, demonstrating the presence of high number of trisomies in the tumor cells (indicated by arrows)

We then performed cytogenetic analysis to determine whether down-regulation of HLTF expression in human colon cancer cells induced chromosomal abnormalities. Detailed SKY analysis of metaphase spreads from both short term (10 days) cultured HCT116 parental cells and HCT116^scramble ^cells revealed the presence of near-diploid chromosomes with a few highly conserved chromosomal translocations (Figure 7A), consistent with the documented karyotype for HCT116 parental cells [37]. This near-diploid karyotype was also observed in the second generation of HCT116 cells and HCT116^scramble ^cells that were derived from the subcutaneous tumors formed by these cells (Figure 7C), further indicating that HCT116 cells have a stable chromosome karyotype as demonstrated previously [38,39]. In contrast, both short-term cultured and the second generation of HCT116^HLTFshRNA1 ^and HCT116^HLTFshRNA2 ^tumor cells exhibited significantly increased chromosomal abnormalities in the forms of chromosomal loss and chromosomal fusions and breaks as compared to the control groups (p < 0.001) (Figure 7B-C). In addition, approximately 15% of metaphases from HCT116^HLTFshRNA1 ^and HCT116^HLTFshRNA2 ^tumor cells showed high numbers of trisomy (Figure 7D). These results clearly indicate that down-regulation of HLTF in human colon cancer cells is able to induce chromosomal abnormalities.

## Discussion

There is now strong evidence that a series of genetic alterations are required for the pathogenesis of human colon cancer. Specific gene mutations, such as in the *APC *gene, initiate the formation of colonic adenomas and others (*e.g. TP53 *mutations as well as other alterations, including KRAS activation) drive the malignant transformation of the adenomas in a multistep progression model [2,3]. More recently, epigenetic alterations, specifically aberrant DNA methylation, have been found to occur commonly in colon cancers [4,5]. Although there is controversy regarding the significance of these alterations in the pathogenesis of colon cancer, more and more data indicate that the aberrant methylation of at least some of these genes, such as *MLH1 *(MutL homolog 1), *MGMT *(O-6-methylguanine-DNA methytransferase) and *HIC1 *(hypermethylated in cancer 1) can be pathogenetic in colorectal carcinogenesis [2,6]. Here, we have added another example to demonstrate the importance of epigenetic alterations in this carcinogenesis.

In this study, we focused on determining the role of loss of HLTF function in the development of colon cancer. The *HLTF *promoter has been found to be hypermethylated in more than 40% of human colon cancers [17-20], suggesting that *HLTF *is a common target for methylation in this cancer. All colon cancer cells that lacked HLTF expression had the methylation of CpG islands within the *HLTF *promoter, while methylation was not detected in the HLTF-expressing cells [17] (Figure 6A), further indicating that this epigenetic event leads to a complete inactivation of HLTF expression in colon cancer cells. To determine the role of loss of HLTF function, in this study, we generated *Hltf *deficient mice (Figure 1). Although these mice did not develop intestinal or colonic tumors, the majority of them were found to develop invasive intestinal and colonic cancers upon Apc mutation-mediated tumor initiation (Figures 4 and 5). Our results strongly suggest that aberrant methylation of HLTF, which leads to the loss of this gene's function as found in most human colon cancers, could have a pathogenetic role rather than being a consequence of colorectal carcinogenesis. This epigenetic event could be important for driving the transition of benign adenomas to the malignant adenocarcinomas, which is consistent with human tumor data showing that *HLTF *promoter methylation is mainly detected in the advanced colonic adenomas and cancers, but not in the early stage of adenomatous polyp [17,19]. The high frequency of *HLTF *promoter hypermethylation observed in these tumors indicates that the silencing of this gene could confer a selective advantage for the progression of these tumors to the malignant cancers.

Although the mechanism by which loss of HLTF function results in this malignant transformation still needs to be defined, our data demonstrating gross chromosomal abnormalities in *Hltf *deficient colon tumor cells may suggest that genomic instability induced in these tumors is a driving force for this carcinogenesis. Genomic instability is a characteristic of human colon cancers, and has been implicated in the initiation and progression of these cancers by increasing the rate of genetic alterations [2]. Two types of genetic instability have been found in human colon cancer: microsatellite instability (MIN) and chromosomal instability (CIN). Tumors with MIN display normal diploid with no obvious chromosomal defects but have high incidence of instability in microsatellite repeats linked to impaired mismatch repair (MMR) [40]. The predominant mechanism identified to inactivate MMR in these tumors is epigenetic silencing through promoter methylation [41], although somatic mutations in MMR genes also occur [42]. CIN, which accounts for 85% of colorectal cancers, exhibits aneuploid, allelic losses and other chromosomal abnormalities, such as translocation, fusion and breaks [32]. Colon cancers with CIN phenotype have been found to behave more aggressively in terms of invasiveness and metastasis [32]. Although its molecular basis remains largely elusive, CIN is thought to arise from structural defects involving centromere or centrosome, microtubule dysfunction, telomere defects, chromosomal fragility and cell cycle checkpoint failure [2,6]. In this work, we have provided solid evidence that *Hltf *deficient mouse colon tumors display severe chromosomal abnormalities, similar to CIN as described in human colon cancers (Figure 5). We have also demonstrated that down-regulation of HLTF increased chromosomal abnormalities in human colon cancer cells (Figure 7). Furthermore, similar to human colon cancers with a CIN phenotype, we have also found that Hltf deficient mouse colon tumors or *HLTF *knockdown human colon cancer cells developed more malignant features, such as invasiveness, as compared to the tumor cells that contain functional HLTF (Figures 4, 5 and 6). All these findings indicate that the genomic instability induced in HLTF deficient colon tumors likely plays an important role in the malignant transformation of these tumors. Future studies using our established *Hltf *knockout mouse colon tumor model to identify the genetic mutations involved in this pathogenesis have the potential to provide a deeper understanding of colon cancer development.

The presence of a CIN phenotype in *Hltf *knockout or knockdown tumor cells also implicates loss of HLTF function as an additional mechanism to induce CIN phenotypes in human colon cancer. The involvement of HLTF in the post-replicative DNA damage-repair pathway has been demonstrated by several recent studies [12-16]. In these work, HLTF was shown to display a similar function as yeast rad5 in the polyubiquitination of PCNA, which is required for initiating an error-free post-replicative repair activity [12,13]. HLTF was also found to have both DNA translocase and chromatin remodeling activities to facilitate the repair of damaged DNA on stalled replication forks [14-16]. Therefore, HLTF could be an important factor in the post-replicative repair pathway. Without HLTF, it is likely that the progression of the replication fork would be blocked at DNA damage lesions, resulting in an accumulation of DNA intermediates that trigger recombination and genomic instability [43]. Our cytogenetic data demonstrating the gross chromosomal abnormalities present in *Hltf *deficient mouse tumor or in HLTF knockdown HCT116 colon cancer cells strongly support this notion. Our data also agree with many other studies indicating that aberrant post-replicative repair activity is a major source of the mutations and chromosome rearrangements that could be important for tumorigenesis. Interestingly, mice deficient in *Hltf *alone were found to develop normally and did not display any pathological disorders associated with genetic instability. This may due to the robust replication checkpoints present in normal mouse cells, which, upon DNA damage, could boost a DNA damage response that arrests cells that harbor unrepaired DNA damage [44]. These unrepaired cells could undergo apoptosis and then be replenished by cells differentiated from a pool of progenitor and stem cells during development. However, when these checkpoint pathways are not functional, the cells that contain defective or unrepaired DNA lesions could still progress through the cell cycle, resulting in broken chromosomes, genome aberrations and an accumulation of mutations [44,45]. A recent study indicates that APC could play an important role in the DNA replication checkpoints by stabilizing the association of DNA replication complexes at stalled DNA replication forks [46]. Consistent with this finding, tumor cells that have lost APC function, such as human SW480 colon cancer cells, have been found to lose the capacity to arrest cells with damaged DNA [46]. Therefore, *Hltf *^-/-^/*Apc^min/+ ^*colon tumor cells may have impaired replication checkpoints, which could facilitate cell cycle progression to enhance the genomic instability in these tumor cells.

HLTF has also been demonstrated to interact with several different gene promoters and enhancers [7-11], suggesting its potential role in the regulation of gene expression. We recently performed micoarray-based gene expression analyses and did not find significant changes of gene expression between *Hltf *^+/+ ^and *Hltf *^-/- ^mouse ES cells or in HCT116 cells with and without HLTF knockdown. In both cases, a very few genes showed 2-fold change with the statistical significance (Additional file 4). None of these genes have been implicated in tumorigenesis or in the maintenance of genomic stability. Therefore, the loss of HLTF function is unlikely to mis-regulate the expression of specific genes, leading to the development of colon cancers.

## Conclusions

We have provided direct genetic evidence that loss of HLTF function promotes the malignant transformation of intestinal or colonic adenomas to carcinomas. We have also demonstrated that this pathogenetic outcome could be associated with the role of HLTF in the maintenance of genomic stability. Given the high frequency of epigenetic inactivation by hypermethylation of HLTF in human colon cancers, our studies strongly suggest that this epigenetic alteration could be directly involved in the development of colon cancer rather than a consequence of this carcinogenesis.

## Methods

### Construction of *Hltf *gene-targeting vector

The *Hltf *gene-targeting vector was generated based on a PCR-based cloning strategy [47]. Briefly, the mouse *Hltf *genomic fragments required for the 5' and 3' arms of homology were PCR-amplified from the genomic DNA of R1 ES cells (on 129S1 background) with a high-fidelity polymerase (TaKaRa). The primer sequences are listed in Additional file 5. After validation by DNA sequencing, the *Hltf 5'arm *DNA fragment was ligated upstream of a *nlsLacZpA*-*lox*P-*pGKneo*-*lox*P cassette. This vector was further inserted by a DNA fragment containing *Hltf 3'arm *and *pGK-DTA *cassette to generate the final gene-targeting vector that contains *Hltf 5'arm-nlsLacZpA-lox*P-*pGKneo-lox*P-*Hltf 3'arm-pGK-DTA *(Figure 1A).

### Generation of *Hltf *deficient mice

The *Hltf *gene-targeting vector was linearized with *Sac*II and then electroporated into R1 ES cells. The transfected ES cells were selected with G418 (250 μg/ml), and G418-resistant ES clones were screened by Southern blot analysis for the correctly targeted allele using *BamH*I (for the 5' external probe) and *EcoR*I (for the 3' external probe) digestions. Both the 5' and 3' external probes were PCR amplified with primers listed in Additional file 5. Two independently targeted ES cell clones were used to generate chimeric mice by ES cell⇔diploid embryo aggregation. Transmitting chimeric males were crossed with an *EIIa-Cre *line (Jackson laboratories, Bar Harbor, Maine) to delete the *lox*P flanked *neo *cassette from the targeted allele. The resultant mouse allele was further bred with 129S1 females to produce hemizygous transgenic offspring on 129S1/C57BL/6 background. *Hltf *^+/- ^mice were also backcrossed to C57BL/6 for eight generations. All mouse experiments were performed in accordance with procedures approved by the University of Manitoba Animal Care and Use Committee.

### Genotyping

A PCR based genotyping method was applied to genotype *Hltf *knockout mice. Primers to amplify the targeted allele were the sense primer (P1) (5'-GGAGCTTTATCAGGCTGTCTGGGA-3') and antisense primer (P2) specific for the *LacZpA *cassette (5'-AGGAAGATCGCACTCCAGCCAGCT-3'). To detect the wild-type *HLTF *allele, wt P1 primer (5'-GTCCATGTCCTAGCCATGAGTA-3') and an antisense primer (P3) locating in exon 1 (5'-GCTTGGTAAGGACTACAAAGCA-3') were used for PCR.

### Mouse intestinal and colonic tumor analysis on *Hltf/Apc *mutant mice

The *Apc^Min/+ ^*mice were purchased from The Jackson Laboratory and maintained on a C57/B6 genetic background. These mice were bred with *Hltf *^+/- ^(C57/B6 background) to generate a cohort of *Hltf *^-/-^/*Apc^min/+^, Apc^min/+ ^*and *Hltf *^+/-^/*Apc^min/+ ^*mice for intestinal tumor analysis. Mice were sacrificed by cervical dislocation, and the entire small intestine and colon were flushed with cold PBS and dissected longitudinally. The number, location and size of tumors in the intestine or colon were examined and recorded. Small intestinal and colonic tumors were further histologically analyzed by hemtoxylin/eosin staining and immunohistochemistry.

### Establishment of colon tumor cells from *Hltf/Apc *mutant mice and growth in *Rag1*^-/-^*/IL2*^-/- ^immunocompromised mice

Tumor cells from *Hltf *^-/-^/*Apc^min/+ ^*and *Apc^min/+ ^*colon tumors were generated as described [35]. The cells were grown in 6 cm culture dishes plated with mitomycin-treated mouse embryonic fibroblast (MEF) cells using the culture conditions as described [35]. The early passage (p2) cells were collected either for tumorigenicity analysis by injecting 10^5 ^cells subcutaneously to *Rag1*^-/-^*/IL2*^-/- ^mice (purchased from Jackson Laboratory), or for cytogenetic characterization.

### Immunohistochemistry (IHC)

IHC was performed as described previously [48]. Briefly, dissected intestinal and colonic tumors were fixed in 10% formalin in PBS overnight. The fixed tissues were embedded in paraffin, and 5 μm paraffin-embedded tissue sections were pretreated with Retrieval solution (Dako) and blocked with either mouse IgG blocking reagent (Vector Laboratory) or serum free blocking reagent (Dako), and then incubated with primary antibodies overnight at 4°. Primary antibodies used were mouse anti-BrdU (Roche, dilution 1:100), mouse anti-Ki67 (BD Pharmingen, dilution 1:100), mouse anti-β-catenin (BD Pharmingen, dilution 1:200), rabbit anti-HLTF (Sigma, dilution 1:100) rabbit anti-lysozyme (Dako, dilution 1:200), and rabbit anti-chromogranin A (ImmunoStar, dilution 1:4000). After washing with TBS containing 0.1% Tween20 buffer, sections were incubated with biotinylated anti-rabbit or anti-mouse antibody for 60 min at room temperature. The sections were washed 3 times with TBS-0.1% Tween20 buffer and incubated with avidin-biotin-peroxidase complex (Vector Laboratory), according to the manufacturer's protocol. Color was developed using metal 3,3'-diaminobenzidine tetrachloride.

### Lentiviral based shRNA knockdown of HLTF expression in HCT116 cells

Lentiviral vectors expressing shRNA against human *HLTF *(NM 003071, V2LHS 254799, _153142, _153143, _153144, _153146) or scrambled control were obtained from the GIPZ lentiviral shRNAmir library (Open Biosystems, Thermo Scientifc) acquired at the University of Manitoba. The pGIPZ lentiviral vector expresses a short hairpin RNA modeled by human microRNA-30 (miR30) primary transcript for increased Drosha and Dicer processing. TurboGFP reporter gene and puromycin drug resistance marker are expressed as part of a bicistronic transcript. Preparation and infection of lentiviral particles into HCT116 cells (from ATCC) were performed based on an established procedure as described previously [49]. After infection, HCT116 cells were treated with 0.75 μg/ml puromycin for four days. The puromycin-resistant clones were pooled to determine HLTF expression by Western blot analysis with anti-HLTF antibody (Sigma). 10^5 ^cells from these established stable cell lines were subcutaneously injected to *Rag1*^-/-^*/IL2*^-/- ^mice to determine the effect of HLTF knockdown on tumor growth. These cells, as well as cells derived from subcutaneous tumors, were also utilized for cytogenetic analysis.

### Cytogenetic analysis

Metaphase spreads for SKY analysis were performed using the SKT kit (ASI) according to the manufacturer's instructions and previously published methods [50]. In each cell line, at least 20 metaphase spreads were studied. Q-FISH analysis was performed as previously described [51].

### Northern blot analysis

Total RNA from freshly dissected mouse E10.5 embryos was extracted using TRIzol (Life Technologies, Inc.). 20 μg of total RNA was separated on a 1% agarose-formaldehyde gel and transferred to Hybond nylon membrane (Amersham). Hybridization was carried out in PerfectHyb (Sigma) with 1.5 × 10^6 ^cpm/ml of 5' and 3' *Hltf *cDNA probes. Both 5' and 3' cDNA probes were PCR-amplified with the primers listed in Additional file 5.

### X-gal staining

Dissected mouse embryos or tissues were fixed for 30 min (for E8.5 to E9.5) or 60 min (for E10.5 embryos and tissues) with 4% paraformaldehyde (PFA), 0.2% glutaraldehyde and 0.02% NP-40 in PBS. Subsequently, fixed samples were washed three times with PBS containing 0.02% NP-40, and stained at 37°C overnight with a staining solution containing 4 mM K_4_Fe(CN)_6_, 4 mM K_3_Fe(CN)_6_, 2 mM MgCl_2_, and 0.2% X-gal in PBS.

### Western blot analysis

For Western blots, cells were lysed in RIPA buffer. 30 μg lysate was separated by 8% SDS-PAGE and transferred to nitrocellulose membrane, which was blocked with 5% non-fat milk and incubated with rabbit anti-HLTF antibody (Sigma, dilution 1:500) at 4° overnight. Protein was detected using an enhanced chemiluminescence system (Amersham Life Science).

### Statistical analysis

A paired *t*-test (two tailed) was used to analyze the statistical significance of chromosomal abnormalities in tumor cells. P < 0.05 was considered indicative of statistical significance.

## Abbreviations

HLTF: Helicase-like Transcription Factor; APC: Adenomatous polyposis coli; PCNA: Proliferating cell nuclear antigen; wt: Wild type; CIN: Chromosomal instability; SKY: Spectral karytotype; MLH1: MutL homolog 1; MGMT: O-6-methylguanine-DNA methytransferase; HIC1: Hypermethylated in cancer 1; MIN: Microsatellite instability; MMR: Mismatch repair; ES: Embryonic stem cells; MEF: Mouse embryonic stem cell; IHC: Immunohistochemistry; PFA: Paraformaldehyde.

## Competing interests

The authors declare that they have no competing interests.

## Authors' contributions

SS, XW, SM and HD conceived and designed the experiments. SS, XW and HD generated the gene-targeting vector and produced the knockout mice and characterized the tumor phenotypes. SS and SM performed cytogenetic analysis. ZN, MR and SK provided reagents and technical supports for this study. SS and HD wrote the manuscript with subsequent contributions from all authors. All authors read and approved the final manuscript.

## Supplementary Material

Additional file 1 **Loss of Hltf function does not affect the cellular proliferation in small intestine and colon**. The small intestines and colons collected from 2-month old *Hltf *^+/+ ^and *Hltf *^-/- ^mice were stained with Haematoxylin-eosin and anti-Brdu antibody. Both *Hltf *^+/+ ^and *Hltf *^-/- ^intestine and colon displayed normal morphology and a similar number of BrdU positive cells within the crypt (2 h after BrdU injection) and in crypt-villus axis (24 h after BrdU injection). Click here for file

Additional file 2 **Loss of Hltf function does not affect the differentiation of epithelial cells in small intestine and colon**. The small intestines and colons from 2-month old *Hltf *^+/+ ^and *Hltf *^-/- ^mice were analyzed using several intestinal cell-lineage markers. The Goblet cells were determined by staining with Alcian blue and periodic acid-Schiff (PAS). The enteroendocrine cells were analyzed by immuno-staining with anti-chromogranin A, and the Paneth cells in small intestine were detected by anti-lysozyme antibody. Both *Hltf *^+/+ ^and *Hltf *^-/- ^intestines or colons showed very similar staining patterns for these markers. Click here for file

Additional file 3 ***Hltf *^-/-^*/Apc^min/+ ^*mice frequently developed invasive intestinal adenocarcinomas**. Several additional Haematoxylin-eosin stained images demonstrate the formation of invasive intestinal adenocarcinomas (indicated by arrows) in *Hltf *^-/-^*/Apc^min/+ ^*mice. Click here for file

Additional file 4 **List of genes that showed expression changes between *Hltf *^+/+ ^and *Hltf *^-/- ^mouse ES cells and in HCT116 cells with and without HLTF knockdown**. The microarray expression assays were carried out by the Center for Applied Genomics at the Toronto Hospital for Sick Children. Three independent cell lines from each group were analyzed. The changes with the statistical significance (p < 0.05) are highlighted by yellow. Red indicates the change of gene expression cannot be validated by real-time PCR. Click here for file

Additional file 5 **List of PCR primers that were applied in this study**. Click here for file
