# Supplementary figures and images for: Loss of HLTF function promotes intestinal carcinogenesis
Source: Mol Cancer. 2012 Mar 27;11:18. doi: 10.1186/1476-4598-11-18 (PMC3337324; doi:10.1186/1476-4598-11-18)

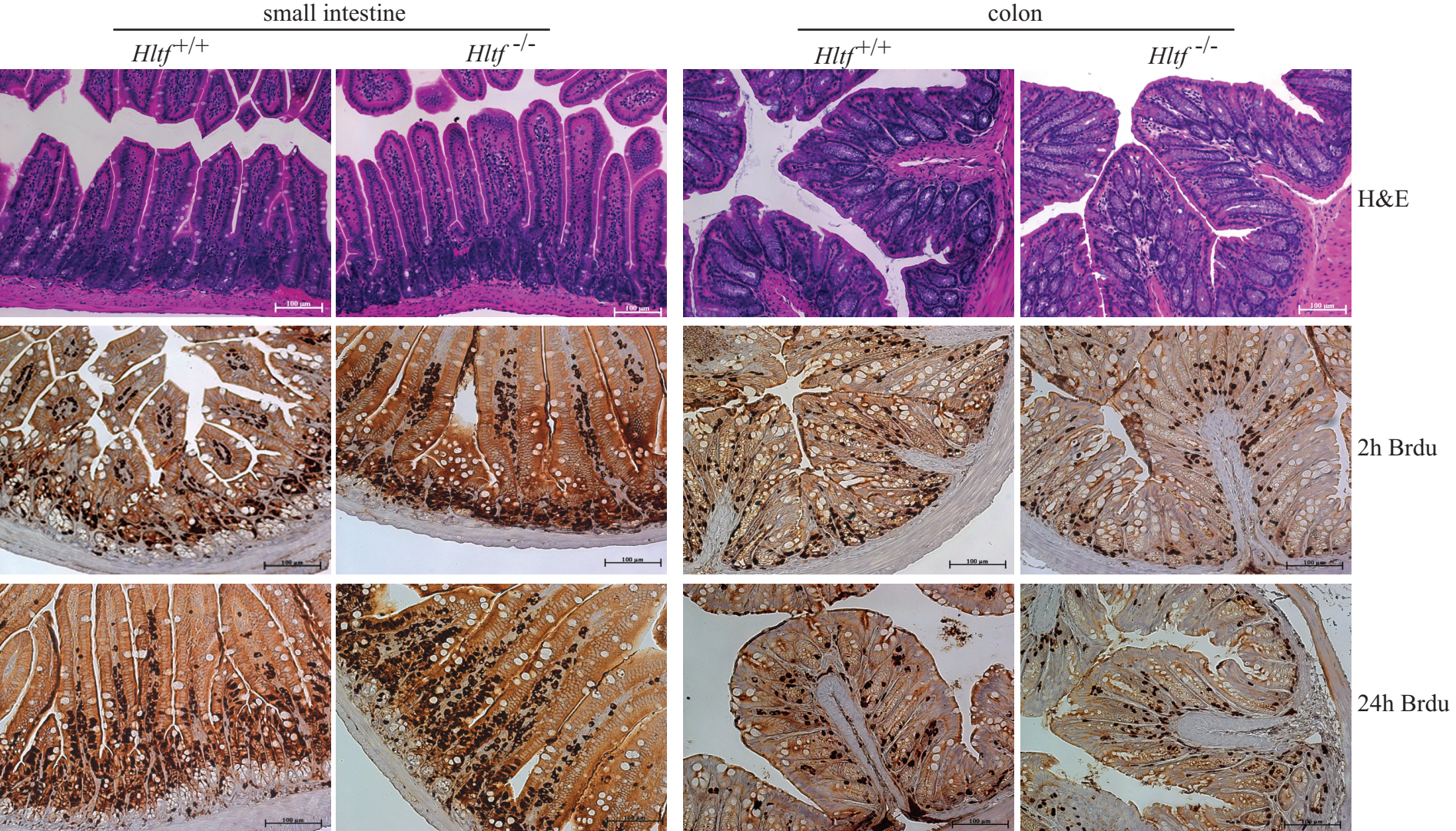

Supplement: Additional file 1 — Loss of Hltf function does not affect the cellular proliferation in small intestine and colon. The small intestines and colons collected from 2-month old Hltf +/+ and Hltf -/- mice were stained with Haematoxylin-eosin and anti-Brdu antibody. Both Hltf +/+ and Hltf -/- intestine and colon displayed normal morphology and a similar number of BrdU positive cells within the crypt (2 h after BrdU injection) and in crypt-villus axis (24 h after BrdU injection). [file 1476-4598-11-18-S1.PDF]

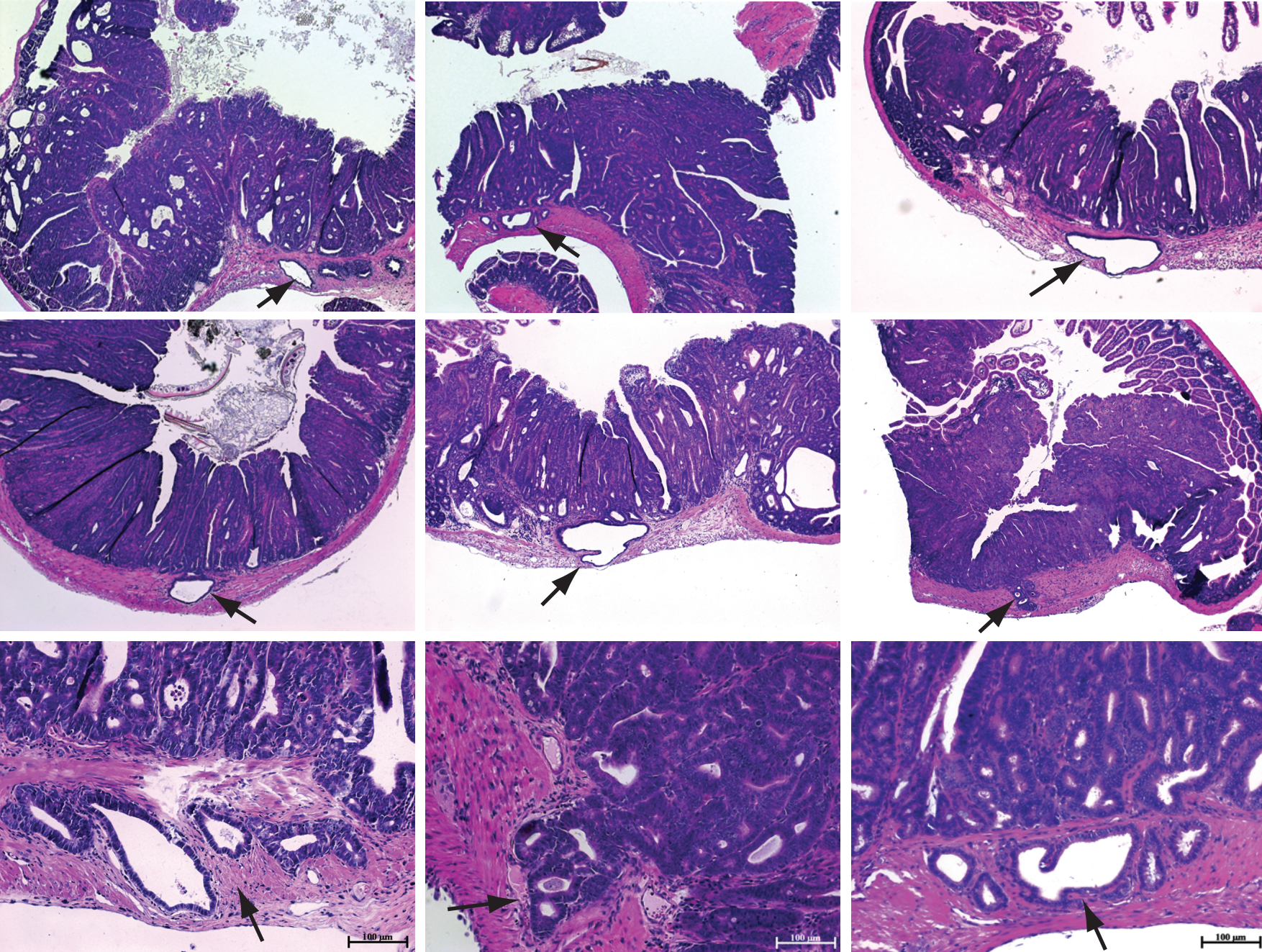

Supplement: Additional file 3 — Hltf -/-/Apcmin/+ mice frequently developed invasive intestinal adenocarcinomas. Several additional Haematoxylin-eosin stained images demonstrate the formation of invasive intestinal adenocarcinomas (indicated by arrows) in Hltf -/-/Apcmin/+ mice. [file 1476-4598-11-18-S3.PDF]
