# Supplementary material for: Loss of HLTF function promotes intestinal carcinogenesis
Source: Mol Cancer. 2012 Mar 27;11:18. doi: 10.1186/1476-4598-11-18 (PMC3337324; doi:10.1186/1476-4598-11-18)
Supplement: Additional file 2 — Loss of Hltf function does not affect the differentiation of epithelial cells in small intestine and colon. The small intestines and colons from 2-month old Hltf +/+ and Hltf -/- mice were analyzed using several intestinal cell-lineage markers. The Goblet cells were determined by staining with Alcian blue and periodic acid-Schiff (PAS). The enteroendocrine cells were analyzed by immuno-staining with anti-chromogranin A, and the Paneth cells in small intestine were detected by anti-lysozyme antibody. Both Hltf +/+ and Hltf -/- intestines or colons showed very similar staining patterns for these markers. [file 1476-4598-11-18-S2.PDF]

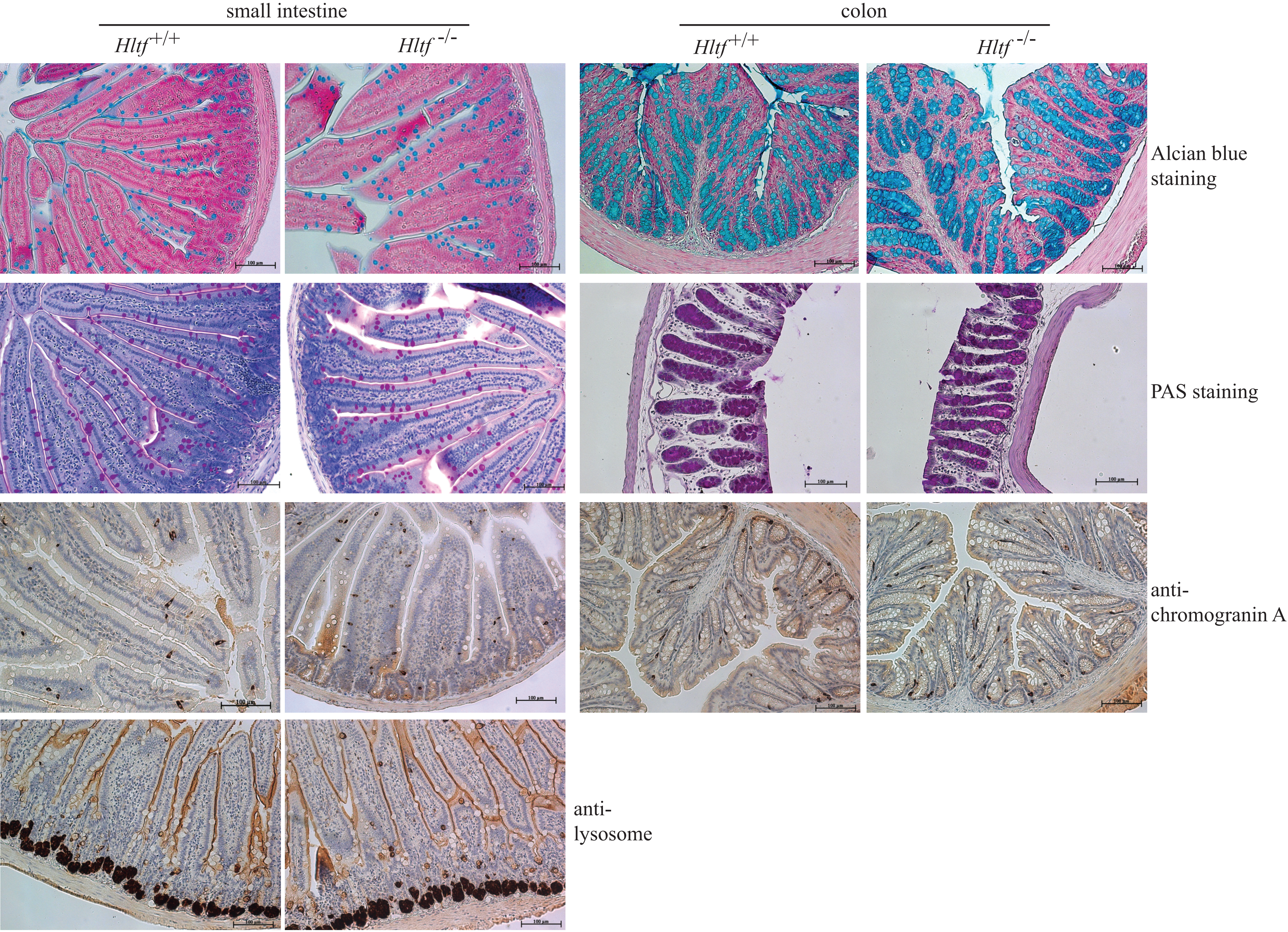

Additional file 2
