# Supplementary material for: Loss of HLTF function promotes intestinal carcinogenesis
Source: Mol Cancer. 2012 Mar 27;11:18. doi: 10.1186/1476-4598-11-18 (PMC3337324; doi:10.1186/1476-4598-11-18)
Supplement: Additional file 4 — List of genes that showed expression changes between Hltf +/+ and Hltf -/- mouse ES cells and in HCT116 cells with and without HLTF knockdown. The microarray expression assays were carried out by the Center for Applied Genomics at the Toronto Hospital for Sick Children. Three independent cell lines from each group were analyzed. The changes with the statistical significance (p < 0.05) are highlighted by yellow. Red indicates the change of gene expression cannot be validated by real-time PCR. [file 1476-4598-11-18-S4.PDF]

Microarray: HLTF homozygous ES cells versus wt ES cells

Column #	Transcript Cluster ID	gene_assignment	Gene Symbol	RefSeq	p-value	Ratio	Fold-Change	Fold-Change Description
4350	10342344	---		---	0.00134393	2.45109	2.45109	homo up vs wt
3043	10341037	---		---	0.0236336	2.18915	2.18915	homo up vs wt
1336	10339330	---		---	0.0238282	0.491285	-2.03548	homo down vs wt
8802	10365627	synaptonemal complex protein 3	Sycp3	NM_011517	0.0512482	0.417201	-2.39692	homo down vs wt
4482	10342476	---		---	0.06096	2.36575	2.36575	homo up vs wt
2976	10340970	---		---	0.092286	2.24911	2.24911	homo up vs wt
24239	10510081	cDNA sequence BC080695	BC080695	BC080695	0.110634	0.462208	-2.16353	homo down vs wt
5615	10343609	---		---	0.126606	2.40587	2.40587	homo up vs wt
29013	10554118	family with sequence similarity 169, member B	Fam169b	NM_001013811	0.131474	0.452159	-2.21161	homo down vs wt
28684	10551236	NLR family, pyrin domain containing 4A	Nlrp4a	NM_172896	0.154665	0.376648	-2.655	homo down vs wt
29791	10560764	predicted gene, EG381936	EG381936	NM_001037248	0.156949	0.409252	-2.44348	homo down vs wt
22492	10494003	TD and POZ domain containing 3	Tdpoz3	NM_207271	0.159113	0.353829	-2.82623	homo down vs wt
29801	10560791	predicted gene, EG381936	EG381936	NM_001037248	0.159712	0.4154	-2.40732	homo down vs wt

Microarray: HLTF shRNA knockdown versus scramble control in HCT116 cells

Column #	Transcript Cluster ID	gene_assignment	Gene Symbol	RefSeq	p-value	Ratio	Fold-Change	Fold-Change Description
22728	8082583	hypothetical LOC442092	ARVP6125	AY358247	0.00202902	0.613491	-1.63001	HLTF shRNA down vs scramble
25612	8110930	---		---	0.00264335	2.61555	2.61555	HLTF shRNA up vs scramble
13347	7987163	formin 1	FMN1	ENST00000414268	0.00274871	1.69517	1.69517	HLTF shRNA up vs scramble
2998	7895511	---		---	0.00946756	2.17754	2.17754	HLTF shRNA up vs scramble
2196	7894705	---		---	0.0115458	2.15453	2.15453	HLTF shRNA up vs scramble
645	7893143	---		---	0.0155399	2.38028	2.38028	HLTF shRNA up vs scramble
2022	7894528	---		---	0.0207031	3.56991	3.56991	HLTF shRNA up vs scramble
23091	8086216	acetyl-CoA acyltransferase 1	ACAA1	AK127051	0.0272952	1.50508	1.50508	HLTF shRNA up vs scramble
113	7892609	---		---	0.0309725	0.374265	-2.6719	HLTF shRNA down vs scramble
3940	7896459	---		---	0.0338883	0.49967	-2.00132	HLTF shRNA down vs scramble
95	7892590	---		---	0.0444153	2.0592	2.0592	HLTF shRNA up vs scramble
1362	7893862	---		---	0.0469824	4.65825	4.65825	HLTF shRNA up vs scramble
5413	7908861	ovarian cancer-related protein 1	OCR1	AF314543	0.0683711	1.50843	1.50843	HLTF shRNA up vs scramble
15175	8006602	chemokine (C-C motif) ligand 4	CCL4	NM_002984	0.0867366	0.641973	-1.5577	HLTF shRNA down vs scramble
