# Supplementary material for: Loss of HLTF function promotes intestinal carcinogenesis
Source: Mol Cancer. 2012 Mar 27;11:18. doi: 10.1186/1476-4598-11-18 (PMC3337324; doi:10.1186/1476-4598-11-18)
Supplement: Additional file 5 — List of PCR primers that were applied in this study. [file 1476-4598-11-18-S5.PDF]

List of PCR primers used in this study

PCR amplification	Forward (5'-3')	Reverse (5'-3')
5’ homology arm	CAGGGTTATACACACAGAGGCCACAAGAAG	CCATGGCGCGGGGCGGGGTGACTTCG
3’ homology arm	ATGAAAAGGAAGGTAGATTCACATATATAC	TTGATTACAGCCATGTTCTGATCTTG
5’ external probe	TCTCTCCACCTGCCCCAACTTGCATCA	CTAGTTCACATCAGGAGTCTCAAGACTGTG
3’ external probe	GCTCTACCCTGGAGATTACATAGATG	GTAAGGTCTAAGGCATTGTCTTCAAAG
5’ cDNA probe	ATACGTTCACGAGGGGTCCTGTTTG	CTGGTCAGTTGTCATCTGTACTGCC
3’ cDNA probe	CAGCCACATGCAAAGTGTCCTTTGTG	GTCATTGGCGTCTGTTTTCTTAGTGC
Real-time PCR for 3’ mouse *Hltf* transcript	TGCGCAGCTTCTCGAGTGTTCTTA	ATCACCTCTTGCTTCTGGCCAAGT
